# 

**Published:** 1874-04

**Authors:** 


					Transactions of the American Dental Association, at the Thir-
teenth Annual Meeting, held at Put-in-Bay, August, 1873.
Chicago, Knight & Leonard, Printers.
The Publication Committee, consisting of Drs. M. S. Dean, G.
H. Cushing and H. A. Smith, can congratulate themselves on
having presented a volume superior in every respect to those
which have preceded it; indeed it is the best ever issued by any
Association.
The Histological illustrations accompanying the report on
Histology, by Dr. T. B. Hitchcock, render this volume of great
value, and well worthy of perusal. Copies of these proceedings
for the year 1873, can be had from the Publication Committee,
550 Michigan Avenue, Chicago. Price on tinted paper, with
Histological illustrations, in muslin covers, $2.00 ; on white pa-
per, without illustrations, in paper covers, $ 1.00.
TO READERS AND CORRESPONDENTS.
Communications intended for publication, and exchange journals and paper
should be addressed to the Editor of the American Journal of Dent./
Science, 259 N. Eutaw St., Baltimore, Md. All letters relating to the busine
of the journal, or containing cash remittances, to be directedto Messrs. Snowdi
& Cowman. Articles for publication should be received by the editor at lea
three weeks previous to the first day of the month on which the number in whic
it is designed for them to appear, is issued.
f^"~Tlie American Journal of Dental Science will contain fifty pages ol
reading matter in each number, making a volume of not less than
Six Hundred pages
EXCLUSIVE OF ADVERTISEMENTS.
T 11E
AMERICAN JOURNAL
OF
DENTAL SCIENCE.
F. J. S. GORGAS, M. D., D.D. S., Editor.
The Journal is issued on the first of each month and contains not less
than fifty pages of reading matter in each number, exclusive of adver-
tisements, making a volume ()i six hundred pages per annum.
In each number will be found Original Communications, Corres-
pondence, Selected Articles, Monthly Summary, Bibliographical No-
tices and Editorial Matter, and every effort will be exerted to render
the Journal worthy of the patronage of the Dental profession.
A generous co-operation i& respectfully solicited from the members
of the profession ; and as the Journal has now a printing office of its
own which materially lessens the cost of its publication, it will be
issued at the reduced subscription price of
$2.SO For Annnm. in Advaxioe.
Communications are respectfully solicited on all subjects pertain-
ing to the practice of dentistry, as well as reports of cases occurring
in dental practice.
RATES OF ADVERTISING.
1 Page.
* "
i "
i ?
1 MO.
$15 00
10 00
' 7 00
5 00
2 MO.
$22 50
15 00
11 00
8 00
3 MO.
$30 00
20 00
15 00
11 00
6 MO.
$50 00
30 00
20 00
15 00
12 MO.
$100 00
50 00
30 00
20 00
All original communications designed for publication, works for
review, new instruments for notice, exchanges, &c., should be directed
to the editor, 259 N. Eutaw St., Baltimpre, Md.
All advertisements and subscriptions should be addressed to
SNOWDEN & COWMAN,
Publishers of the Am. Journal of Dental Science.
86 W. Fayette St. Bet. Charles & Liberty Sts.,
BALTIMORE, Md.

				

